# The Black-Box of Plant Apoplast Lipidomes

**DOI:** 10.3389/fpls.2016.00323

**Published:** 2016-03-18

**Authors:** Biswapriya B. Misra

**Affiliations:** Department of Biology, Genetics Institute, University of FloridaGainesville, FL, USA

**Keywords:** fatty acids, lipid transfer protein, lipidomics, xylem sap, secretome

## Introduction

The apoplast has gained recent attention owing to advances in—omics approaches, esp. the proteomics studies in apoplasts, xylem saps, and intercellular washing fluids (IWF) from diverse plant species. Apoplastic interactions are integral to plant signaling, growth, defense, physiology, and reproduction. In addition, the plant apoplast serves as a hub of pathogen effectors and a great deal of pathogenic proteins and small molecules are cataloged in literature. In spite of its importance in plant biology, the knowledge about apoplastic lipids and their carriers have been limited to few seminal studies and sporadic efforts. Here, the attempt is to comprehend the gained knowledge and remaining gaps in plant apoplastic lipidomes and address means as to how efforts can unveil the black box of plant apoplastic lipidome. The apoplast is defined as the extracellular matrix, the plant cell wall and the intercellular spaces where the apoplastic fluid circulates (Agrawal et al., 2010). In addition, the intercellular fluids and xylem sap samples, for instance represent the apoplastic system that transports distinct molecules along the plant system (Kehr and Rep, 2007; Seifert and Blaukopf, 2010). In vascular plants, the apoplastic xylem saps demonstrate considerable differences in composition from the apoplast used in phloem loading. The complement of all proteins and metabolites that are exported out of the symplast comprises the plant's secretome (or apoplastic proteome and metabolome, respectively). Important roles assigned to apoplastic proteome is that of conferring basal immunity (Feussner and Polle, 2015) among other important functions. Plant apoplast proteomics studies conducted in tobacco (Dani et al., 2005; Goulet et al., 2010), cowpea (Fecht-Christoffers et al., 2003), *Arabidopsis* (Boudart et al., 2005; Kwon et al., 2005; Ge et al., 2011; Floerl et al., 2012), canola (Floerl et al., 2008), rice (Zhang et al., 2009), soybean (Djordjevic et al., 2007), poplar (Pechanova et al., 2010), and *Medicago* (Soares et al., 2009) have furthered our knowledge by helping researchers catalog proteins involved in pathogen interactions, heavy metal accumulation, oxidative stress, pollen germination, cell wall biosynthesis and regeneration, salinity stress tolerance among other pivotal plant physiological, and defense responses. Moreover, the plant secretome proteomes have been reviewed elsewhere (Jwa, 2008; Agrawal et al., 2010; Alexandersson et al., 2013; Krause et al., 2013). In contrast, transcriptomic (Blomster et al., 2011) and metabolomics (Floerl et al., 2012) studies of apoplasts are rare and remains to be explored.

Surprisingly, absolutely no information is available yet on the lipidome of the plant apoplast. Plant lipidome is huge and the major classes of lipids discovered till date include, but are not limited to, triacylglycerols (TAGs)—the most abundant class of storage lipids, wax esters, sterols, sterol esters, acylated sterol glycosides, phytoglycolipids, ceramides, glucosylceramides. For instance only phospholipids consists of major classes of phospholipids such as phosphatidylcholines (PC), phosphatidylglycerols (PG), phosphatidylethanolamines (PE), phosphatidylserines (PS), and phosphatidylinositols (PI) are structural phospholipids that have distinct and specific distributions in the cellular membranes, contributing to their identity (van Meer et al., 2008). On the other hand, phosphatidic acid (PA), phosphatidylinositolmonophosphate (PIP), phosphatidylinositolbisphosphate (PIP_2_) and lysophospholipids such as lysophosphatidylcholine (LPC), lysophosphatidylethanolamine (LPE), and lysophosphatidic acid (LPA) are produced from structural phospholipids by specific enzymatic pathways and are minor constituents of the cell membranes (Meijer and Munnik, 2003). Although information on the plant lipidome exists in the form of oil-crops and seed oils, little is known about their apoplastic abundances or distribution.

Hence, in this review, we look into the apoplast lipids and their associated biosynthetic machineries, where a lot have been deciphered in terms of bound proteins, polymers, and proteins. Apoplast includes the cell-walls which are known to boast a diverse metabolites, the extracellular spaces where the “secretomes” are released (including the organ surfaces) and cuticles which is consisted of lipid and hydrocarbon polymers impregnated with wax among others. Cell walls include macromolecular polymeric structures such as lignins, pectins, cellulose among others. Suberin is an apoplastic biopolymer that contributes to the control of diffusion of water and solutes across internal root tissues and in periderms (Ranathunge et al., 2011). However, I exclude the cell-wall bound apoplastic constituents to focus on the dynamic aspects of lipidome.

## Apoplastic proteins involved in transfer of lipids

Lipids, are extremely hydrophobic polymer compounds that have to pass through the apoplastic compartment or eventually to the highly hydrophilic cell wall for incorporation. Thus, it has been deciphered that non-specific lipid transfer proteins (nsLTPs) are secreted into the apoplast, that contain a hydrophobic pocket which binds long-chain fatty acids (FA) to transfer lipids via an unknown mechanism (Edstam and Edqvist, 2014). In one of the pioneering and seminal investigation, in *Arabidopsis thaliana* it was observed that a *defective* in *induced resistance 1-1* (*dir1-1*) mutant exhibited wild-type local resistance to avirulent and virulent *Pseudomonas syringae*, failed to develop systemic acquired resistance (SAR) to virulent pathogens such as *Pseudomonas* or *Peronospora parasitica* (Maldonado et al., 2002). Thus, *DIR1* was shown to encode a putative apoplastic LTP which interacts with a lipid-derived molecule to promote long distance signaling during pathogenesis (Maldonado et al., 2002). Following this work, there have been numerous instances where LTPs were simply reported or their functional roles were probed. nsLTPs are localized extracellularly in barley, carrot, grape, *Arabidopsis*, tobacco, soybean, *Medicago* (Liu et al., 2015), while proteomics studies have indicated their sub-cellular localizations in apoplastic fluids (Dani et al., 2005; Djordjevic et al., 2007). Other efforts in this direction, which have also included similar proteins that are involved in apoplastic lipid transport or metabolism, are summarized below.

In *Euphorbia lagascae* seedlings, *E. lagascae* lipid transfer proteins (ElLTP2) function as a apoplastic carrier when lipid components from the senescent cells of the endosperm (Eklund and Edqvist, 2003). They observed that ElLTP2 are relocalized to the growing cotyledons where lipids are used in epidermal growth and development. In the xylem-sap of tomato, a new family of small cysteine-rich proteins (XSP10), with structural similarity to LTPs was reported (Rep et al., 2003). The declined protein levels of XSP10s in tomato plants infected with a fungal vascular pathogen possibly indicated its breakdown or modification by the pathogen (Rep et al., 2003). The *ATT1* (for *a*berrant induction of *t*ype three genes, encodes a CYP86A2), a cytochrome P450 monooxygenase catalyzing fatty acid oxidation was reported in the apoplast (Xiao et al., 2004). This protein was shown to be involved as a major player in biosynthesis of extracellular lipids and cutin biosynthesis. nsLTPs were also detected in apple (*Malus domestica* cv. Elstar) leaves infected with a cloned isolate of the apple scab *Venturia inaequalis* (Gau et al., 2004). When the researchers collected the IWF from the uninfected leaves, the protein was detected but its amount declined to a non-detectable level within the 1st week post-infection. Furthermore, in tobacco leaves subjected to salt stress, two LTPs were expressed entirely *de novo* (Dani et al., 2005). GLIP1, is a secreted lipase with a GDSL-like motif designated GDSL LIPASE1 is salicylic acid dependent (Oh et al., 2005). In addition to exhibiting antimicrobial activity, the *glip1* plants were shown to be compromised in local and systemic resistance to the necrotrophic pathogen *Alternaria brassicicola.* Other LTPs were also reported in the xylem apoplast of *Glycine max* (Djordjevic et al., 2007). Similarly, Ha*AP10*, an LTP from *Helianthus annuus* dry seeds, is apoplastic in dry seeds and upon imbibition is rapidly internalized and relocalized to organelles involved in lipid metabolism—acting as a fatty acid shuttle between the oil body and the glyoxysome during seed germination (Pagnussat et al., 2012). Phospholipase A_2_ (PLA_2_) hydrolyzes phospholipids at the *sn*-2 position to yield lysophospholipids and free fatty acids. With leaf maturity and upon pathogenic challenge the paralog PLA_2_α is translocated from ER/Golgi locations to apoplastic space (Jung et al., 2012). A glycophosphatidylinositol (GPI)-linked lipid transfer protein (LTPG) accumulates specifically at junctional borders, i.e., resides both in the apoplastic space between the plasma membrane, cell wall, and in the intercellular fluids (Ambrose et al., 2013).

Thus, the nsLTPs, LTPs and associated proteins indicate a dynamic role of these proteins in lipid biosynthesis, transport, and metabolism in the plant apoplast—be it, in the seeds, infested leaves, or during a diverse stress conditions.

## Lipids that are secreted into apoplast

**Figure 1:**
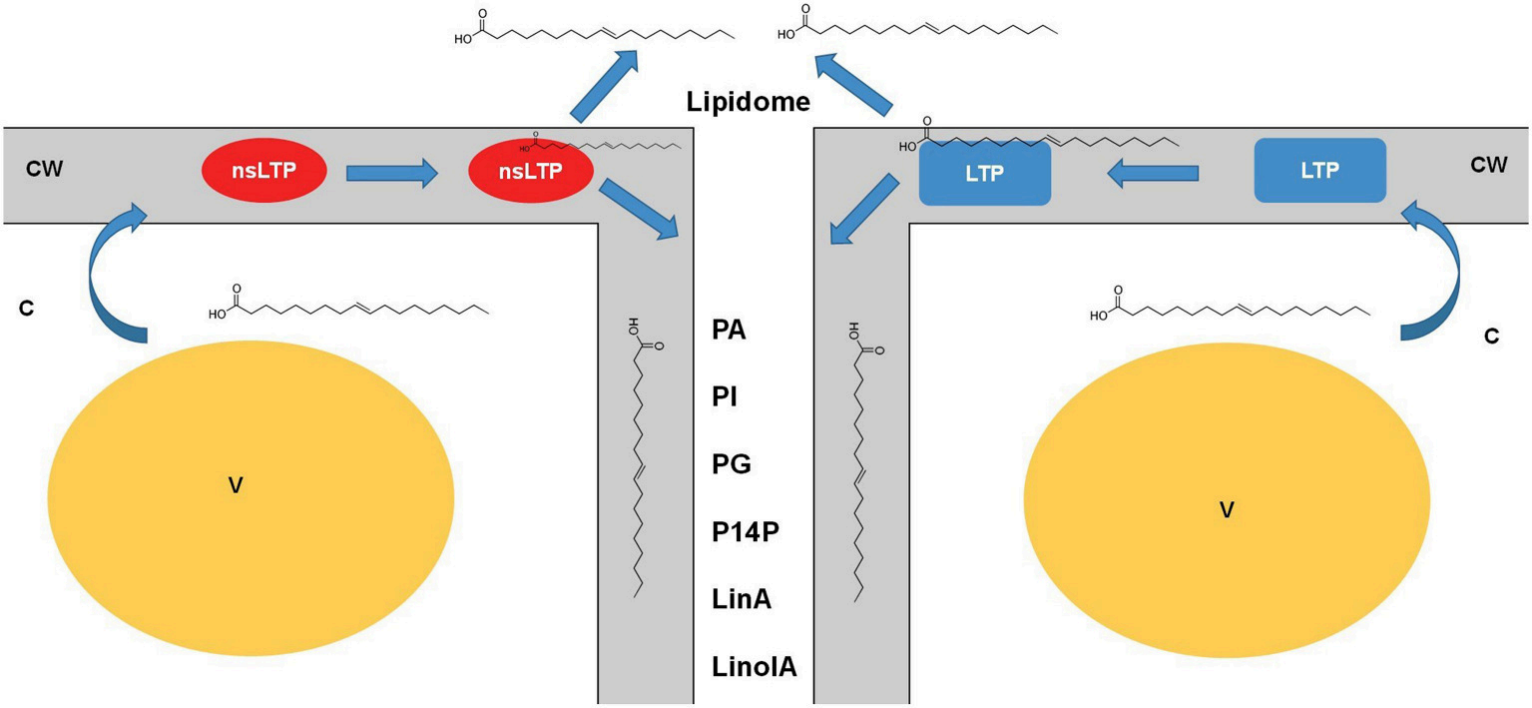
**Dynamic role of lipid transfer proteins in plant apoplast**. The lipid transfer proteins such as LTPs and nsLTPs are possibly the major players in carrying the diverse lipid classes from the cytosol (symplast) through the cell wall to apoplast. Little information is established as to how the turnover of these proteins, involvement of other players, and mechanisms of loading/ unloading happen to generate this apoplastic lipidome. Nonetheless, with lack of concrete evidence it is safe to speculate that these LTPs are major players in apoplastic lipid metabolism. Cataloging of these lipids using mass-spectrometry platforms would be insightful in understanding the real time kinetics of such transfers across the membranes. nsLTPs, non-specific lipid transfer proteins; LTP, lipid transfer proteins; CW, Cell wall; V, vacuole; C, cytoplasm; LinA, linoleic acid; LinolA, linolenic acid; PA, phosphatidic acid; PI, phosphatidylinositol; PG, phosphatidylglycerol; PI4P, phosphatidylinositol 4-phosphate.

Suberin is a heteropolymer with polymeric aliphatic and associated aromatic materials. Long-chain oxygenated fatty acids are the core constituents of the suberin polyester. *In* oats (*Avena sativa*) the apoplastic (exoplasmic) leaflet, as well as in rafts, phospholipids did not include digalactosyldiacylglycerol (DGDG), but showed the presence of acylated sterol glycosides (Tjellström et al., 2010). Moreover, they observed that this phospholipid accumulation was observed to be dependent on the phosphate availability status of the plants. The extracellular washing fluids (EWFs) obtained from sunflower (*Helianthus annuus*) seeds imbibed for 2 h contained diverse phospholipids, i.e., PA and PI being the major phospholipids (Regente et al., 2008). In addition, the phytohormone abscisic acid (ABA) and jasmonic acid (JA) induced changes in phospholipid profiles, i.e., JA—induced decreased in PI and increased PA, and ABA—induced increased in PA and PG indicate their contribution in intercellular communication. Further, important plant lipid signaling components such as phosphatidylinositol-4-phosphate (PI4P) were reported in the extracellular medium of tomato cell suspensions as well as in the apoplastic fluids of tomato plants (Gonorazky et al., 2012). In addition to a diverse phospholipids, electron spray ionization—tandem mass spectrometry (ESI-MS/MS) analysis indicated a markedly different profile from the phospholipid pattern identified in entire leaflets. Moreover, the levels of each phospholipid detected in entire leaflets were at the nanomolar scale, while the levels of the distinct apoplastic phospholipids were at the picomolar scale. In French bean (*Phaseolus vulgaris*) leaf cells, the characteristic oxylipins for oxidation were not detected in apoplastic fluid over the period of the apoplastic burst, although linolenic and linoleic acids were detected in apoplastic fluids before elicitation (Bolwell et al., 2002). With a handful of lipids and fatty acid metabolites recorded in the apoplast, it remains to be seen, if these revelations are biologically relevant or it is just the case of lack of structured studies into the lipid classes of apoplasts. In Figure 1, the available knowledge on the LTPs and handful of lipids for which very little functions are established, are summarized.

## Conclusions and future perspectives

We have just started exploring and understanding the apoplastic lipidomes at the levels of singular carrier proteins only, i.e., more specifically the LTPs and their functionalities in pathogenesis and stress conditions, mostly. Only a handful of lipids have been localized to the apoplast or roughly assigned any function in the apoplast. Thus, we have only touched the tip of the ice berg of the entire plant lipidome which must be dynamic and diverse. However, with expanding lipidomic studies in model systems such as *Arabidopsis* (Degenkolbe et al., 2012; Higashi et al., 2015), maize (Riedelsheimer et al., 2013), oil crops (Furse et al., 2013), tobacco (Li et al., 2015) and microbial systems such as cyanobacteria (Plohnke et al., 2015), and yeast (Ejsing et al., 2009; Grillitsch et al., 2011), single cell-types such as oleaginous algae (Li et al., 2014), organellar (Rolland et al., 2009), and sub-cellular (Horn and Chapman, 2012) domains, our understanding of the lipidome (Welti et al., 2007) is constantly gaining newer insights for bioprospecting of lipids in medicine, biotechnology, food, pharmaceuticals, agriculture, and biofuels. In addition, the newer technologies available to address plant lipidome that span from *in situ* lipidomic visualization/ imaging (Horn and Chapman, 2014), shotgun lipidomics (Han and Gross, 2005), hydrophobic interaction liquid chromatography—ion trap—time of flight- mass spectrometry (HILIC-IT-ToF-MS) (Okazaki et al., 2013), to ultra-pressure liquid chromatography—high resolution mass spectrometry (UPLC-HRMS) (Hummel et al., 2011) and associated development in softwares/ tools for analyses of lipidomic data sets (Haimi et al., 2006); we are at exciting times of looking at plant apoplastic lipidomes with a rejuvenated interest.

## Author contributions

The author confirms being the sole contributor of this work and approved it for publication.

### Conflict of interest statement

The author declares that the research was conducted in the absence of any commercial or financial relationships that could be construed as a potential conflict of interest.
